# The HTT1a protein initiates HTT aggregation in a knock-in mouse model of Huntington’s disease

**DOI:** 10.1093/brain/awag040

**Published:** 2026-02-02

**Authors:** Aikaterini Smaragdi Papadopoulou, Christian Landles, Edward J Smith, Marie K Bondulich, Annett Boeddrich, Maria Canibano-Pico, Emily C E Danby, Franziska Hoschek, Arzo Iqbal, Samuel T Jones, Nancy Neuendorf, Iulia M Nita, Georgina F Osborne, Jemima Phillips, Maximilian Wagner, Erich E Wanker, Jonathan R Greene, Andreas Neueder, Gillian P Bates

**Affiliations:** Huntington’s Disease Centre and Department of Neurodegenerative Disease, Queen Square Institute of Neurology, UCL, London WC1N 3BG, UK; Huntington’s Disease Centre and Department of Neurodegenerative Disease, Queen Square Institute of Neurology, UCL, London WC1N 3BG, UK; Huntington’s Disease Centre and Department of Neurodegenerative Disease, Queen Square Institute of Neurology, UCL, London WC1N 3BG, UK; Huntington’s Disease Centre and Department of Neurodegenerative Disease, Queen Square Institute of Neurology, UCL, London WC1N 3BG, UK; Neuroproteomics, Max Delbrueck Center for Molecular Medicine, Berlin 13125, Germany; Huntington’s Disease Centre and Department of Neurodegenerative Disease, Queen Square Institute of Neurology, UCL, London WC1N 3BG, UK; Huntington’s Disease Centre and Department of Neurodegenerative Disease, Queen Square Institute of Neurology, UCL, London WC1N 3BG, UK; Department of Neurology, University Hospital Ulm, Ulm 89081, Germany; Huntington’s Disease Centre and Department of Neurodegenerative Disease, Queen Square Institute of Neurology, UCL, London WC1N 3BG, UK; Huntington’s Disease Centre and Department of Neurodegenerative Disease, Queen Square Institute of Neurology, UCL, London WC1N 3BG, UK; Neuroproteomics, Max Delbrueck Center for Molecular Medicine, Berlin 13125, Germany; Huntington’s Disease Centre and Department of Neurodegenerative Disease, Queen Square Institute of Neurology, UCL, London WC1N 3BG, UK; Huntington’s Disease Centre and Department of Neurodegenerative Disease, Queen Square Institute of Neurology, UCL, London WC1N 3BG, UK; Huntington’s Disease Centre and Department of Neurodegenerative Disease, Queen Square Institute of Neurology, UCL, London WC1N 3BG, UK; Department of Neurology, University Hospital Ulm, Ulm 89081, Germany; Neuroproteomics, Max Delbrueck Center for Molecular Medicine, Berlin 13125, Germany; Rancho BioSciences, San Diego, CA 92127, USA; Department of Neurology, University Hospital Ulm, Ulm 89081, Germany; Center for Molecular Neurobiology, University Medical Center Hamburg-Eppendorf, Hamburg 20251, Germany; Huntington’s Disease Centre and Department of Neurodegenerative Disease, Queen Square Institute of Neurology, UCL, London WC1N 3BG, UK

**Keywords:** *HTT* transcription, HTT lowering, HTRF, transcriptional dysregulation, NEFL/BRP39/YKL40 biomarkers

## Abstract

The mutation that causes Huntington’s disease is a CAG repeat expansion in exon 1 of the huntingtin gene (*HTT*) that leads to an abnormally long polyglutamine tract in the huntingtin protein (HTT). Mutant CAG repeats are unstable and increase in size in specific neurons and brain regions with age, a phenomenon that constitutes the first step in the pathogenesis of the disease. In the presence of an expanded CAG repeat, cryptic polyadenylation (polyA) sites in intron 1 of the *HTT* pre-mRNA can become activated leading to the polyadenylation of a prematurely terminated transcript, *HTT1a*. This encodes the HTT1a protein, which is known to be very aggregation-prone and highly pathogenic. Given that the longer the CAG repeat the more HTT1a is generated, could the production of HTT1a be the mechanism through which somatic CAG repeat expansion exerts its pathogenic consequences? Resolving this issue is very important for the design of therapeutic approaches to lower huntingtin levels.

We have used a clustered regularly interspaced short palindromic repeats (CRISPR)-Cas9 approach to prevent the production of HTT1a in a knock-in mouse model of Huntington’s disease. All potential cryptic polyA sites were deleted from *Htt* intron 1 in *Hdh*Q150 mice and colonies were established that were heterozygous for the intron 1 deletion on a mutant allele (*Hdh*Q150ΔI) and heterozygous for the deletion on a wild-type allele (WTΔI). The CAG repeat sizes in the *Hdh*Q150 and *Hdh*Q150ΔI colonies were well-matched at approximately 195 CAGs.

As predicted, the deletion of the cryptic polyA sites from *Htt* intron 1 prevented the generation of the *Htt1a* transcript in the *Hdh*Q150ΔI mice. However, very low levels of the HTT1a protein were detected, which resulted from a *Htt readthrough* product of exon 1 and exon 2, that had retained the deleted intron and terminated at a cryptic polyA site in intron 2. *Hdh*Q150, *Hdh*Q150ΔI, wild-type and WTΔI mice were studied until 17 months of age. Immunohistochemical and homogeneous time-resolved fluorescence analysis showed that HTT aggregation in both *Hdh*Q150 and *Hdh*Q150ΔI brains contained HTT1a, but the dramatic decrease in soluble HTT1a levels in *Hdh*Q150ΔI brains delayed the appearance of aggregated HTT1a by several months. Although this delay in aggregate pathology only partially reversed transcriptional dysregulation, the biomarkers neurofilament light polypeptide (NEFL) and breast regression protein 39 (BRP39) (YKL40) remained at wild-type levels in *Hdh*Q150ΔI mice at 17 months of age.

These data demonstrate that the production of HTT1a initiates HTT aggregation and that it is important to target *HTT1a* in huntingtin-lowering therapeutic strategies.

## Teaser

Papadopoulou *et al.* used CRISPR to block production of the HTT1a protein in a knock-in mouse model of Huntington’s disease. This intervention delayed the appearance of aggregates in the brain by several months and maintained CSF disease markers at normal levels, supporting therapeutic strategies aimed at reducing HTT1a levels.

## Introduction

Huntington’s disease is an inherited neurodegenerative disorder with symptoms that include dysregulated movements, psychiatric disturbances and cognitive decline.^1^ It is caused by a CAG repeat expansion in exon 1 of the huntingtin gene (*HTT*) that encodes a polyglutamine tract in the huntingtin protein (HTT).^2^ Individuals with repeats of (CAG)_35_ or less do not develop Huntington’s disease and a repeat of (CAG)_40_ or more, as measured in blood, is a fully penetrant mutation.^3^ The length of the mutant CAG repeat partially accounts for the age of disease onset, with mutant alleles of (CAG)_≥60_ often causing onset of symptoms in childhood.^4^ The neuropathology of Huntington’s disease involves neuronal cell death in the striatum, cortex and other brain regions,^5,6^ and HTT inclusions can be identified in neuronal nuclei and scattered throughout the neuropil.^5,6^

The human *HTT* gene contains 67 exons, and the mature transcript is translated to produce a large multifunction scaffold protein.^7^ An expanded CAG repeat can result in the alternative processing of the *HTT* pre-mRNA through the activation of cryptic polyadenylation (polyA) sites and the termination of transcription in intron 1.^8,9^ The cryptic polyA sites are located at 2710 and 7327 base pairs (bp) into intron 1 in human *HTT* and 680 and 1145 bp into intron 1 in mouse *Htt*.^8,9^ As the CAG repeat increases in size, the extent to which the huntingtin pre-mRNA is processed to generate *HTT1a* or *Htt1a* increases.^8,10^ The human *HTT1a* and mouse *Htt1a* transcripts encode the exon 1 HTT protein (HTT1a) that terminates in a proline residue, and does not contain any amino acids that are not present in the full-length HTT protein.^8^

The R6 lines of mice are transgenic for a genomic construct that contains human *HTT* promoter sequences, exon 1 and ∼220 bp of intron 1.^11^ The transcription of this transgene terminates at cryptic polyA sequences in the mouse genome close to the integration sites; therefore, the R6 lines of mice express the human HTT1a protein. Analysis of R6 lines has demonstrated that HTT1a is highly pathogenic; expression of the transgene with a (CAG)_∼120_ in R6/2 mice, at similar levels to a wild-type (WT) *Htt* allele, resulted in end-stage disease at ∼12 weeks of age.^11^ Over the past 25 years multiple studies expressing a mutant version of HTT1a have confirmed that it is aggregation-prone and very pathogenic in biochemical assays and multiple models including cells in culture, fruit flies, mice, rats and non-human primates.^12^

Somatic CAG repeat expansion can result in repeats containing several hundred CAGs in specific brain regions and cell types in people with Huntington’s disease.^13-17^ Genome-wide association studies (GWAS) for genetic modifiers of Huntington’s disease phenotypes have identified six genes that play a role in DNA mismatch repair.^18-23^ These modifiers most likely act by delaying somatic CAG repeat expansion, given that the ablation of specific mismatch repair genes in mouse models of Huntington’s disease prevents somatic CAG repeat instability.^24-26^ It is now widely accepted that somatic CAG repeat expansion represents the first step in the molecular pathology of Huntington’s disease.^27^ Given the pathogenic nature of HTT1a, and that its levels increase with increasing CAG repeat length, could its production be the next step in the pathogenic cascade?

In this study, we have used a genetic strategy aimed at preventing the generation of HTT1a in a knock-in mouse model of Huntington’s disease. By deleting the cryptic polyA sites from *Htt* intron 1 in the *Hdh*Q150 knock-in mouse model, the level of the HTT1a protein was considerably depleted. The deposition of HTT aggregation in brain regions was significantly delayed, transcriptional dysregulation was improved, and plasma and CSF biomarkers were maintained at WT levels. These data demonstrate that the HTT1a protein initiates HTT aggregation and is a major contributor to Huntington’s disease phenotypes.

## Materials and methods

### Mouse breeding and maintenance

All procedures were performed in accordance with the Animals (Scientific Procedures) Act, 1986 and approved by the University College London Ethical Review Process Committee. Mouse husbandry and health monitoring were as previously described.^28^ Animals were kept in individually ventilated cages with Aspen Chips 4 Premium bedding (Datesand) and environmental enrichment comprising chew sticks and a play tunnel (Datesand). All mice had constant access to water and food (Teklad global 18% protein diet, Envigo). Temperature was regulated at 21°C ± 1°C and mice were kept on a 12-h light/dark cycle. The facility was barrier-maintained and quarterly non-sacrificial FELASA (Federation of European Laboratory Animal Science Associations) screens found no evidence of pathogens.

### Generation of the *Hdh*Q150ΔI and WTΔI mouse colonies

The *Hdh*Q150ΔI and WTΔI mice were generated at SAGE Labs (Horizon Discovery). Briefly, *Hdh*Q150 heterozygous males were bred to superovulated C57BL/6J females (Jackson Laboratory), and fertilized embryos were microinjected with single guide RNAs (sgRNAs) and Cas9. Given that 50% of the embryos would be heterozygous for the mutant allele and 50% would be wild type, 75% of the alleles available for modification were wild type. The upstream sgRNA was tctagggttacctcctcatcagg and the downstream sgRNA was tgtgttaggcaaattttggtggg. DNA was extracted from ear biopsies of the progeny using the Epicentre QuickExtract solution (Cambio). Genotyping was in Sigma JumpStart Taq ReadyMix (P2893) with 1 µM primers for 5 min at 95°C, 35× (30 s at 95°C, 30 s at 60°C, 1 min at 68°C), 5 min at 68°C. The forward and reverse primers flanking the upstream sgRNA site were: F1 = tccctcagaggagacagagc, R1 = cattggatgcgttcacactc. The forward and reverse primers flanking the downstream site were: F2 = cattgctgcagtgagcttct, R2 = gcttcctcacacgacactca. Non-homologous end-joining efficiency at the upstream site (F1-R1) was 53% and at the downstream site was 15% (F2-R2). The efficiency of the intronic deletion between the sgRNAs was 4.3% (F1-R2). Of 162 pups screened, seven founders with a deletion on the WT allele were identified and the PCR band extracted and sequenced (Supplementary Table 1). Colonies were established for founders #123 and #146 by backcrossing to C57BL/6J (Charles River) and line 123 was used as the WTΔI line described in this manuscript.

The experiment was repeated and to increase the chance of generating the deletion on the mutant allele, *Hdh*Q150 males were bred to superovulated homozygous WTΔI females, generating embryos in which 50% of the alleles available for modification were mutant. The upstream sgRNA was tctagggttacctcctcatcagg, as before, and the downstream sgRNA was shortened to tgttaggcaaattttggtggg. Genotyping revealed that non-homologous end-joining efficiency at the upstream site was 34.1% and at the downstream site was 30%. From 110 pups, five founders were recovered with the deletion on both WT alleles, and one male founder (#66) with a 20 014 bp deletion on the mutant allele (genomic coordinates 716–20 729). A colony was established for founder #66 (*Hdh*Q150ΔI) by backcrossing to C57BL/6J females (Charles River).

### Genotyping and CAG repeat sizing

Genomic DNA isolation from ear biopsies was as previously described.^29^ To genotype for the CAG repeat mutation in the *Hdh*Q150 and *Hdh*Q150ΔI mice, the forward and reverse primers were: MHD16 CCCATTCATTGCCTTGCTGCTAAG and MHD18 GACTCACGGTCGGTGCAGCGGTTCC. DNA (1.2–2.4 ng DNA/µl) was amplified in AmpliTaq Gold 360 master mix (Thermo Fisher Scientific) with GC enhancer (Thermo Fisher Scientific) and 0.4 µM each primer at 5 min at 95°C, 30× (30 s at 94°C, 30 s at 58°C, 3 min at 72°C), 5 min at 72°C. For CAG repeat sizing, 1.2 ng/µl was amplified with a fluorescein amidite (FAM)-labelled reverse primer. To genotype for the intron 1 deletion in the *Hdh*Q150ΔI and WTΔI mice the forward primers were: Din1-03 TCGTCTTGCGGGGTCTCTGG (WT allele) and Din1-05 TTCTGACCGGTCACGTAAACTGCT (mutant allele) and the reverse primer was Hdh009Rv CACACCTAAGTAACTCACCATGTACTTG. DNA (2.4 ng) was amplified in AmpliTaq Gold 360 master mix (Thermo Fisher Scientific) with GC enhancer (Thermo Fisher Scientific) and 0.4 µM each primer at 2 min at 94°C, 35× (15 s at 94°C, 20 s at 62°C, 45 s at 72°C), 3 min at 72°C. The CAG repeat size of the *Hdh*Q150 colony was 195.76 ± 6.95 and of the *Hdh*Q150ΔI colony was 199.87 ± 9.61.

### Antibodies

The antibodies used for homogeneous time-resolved fluorescence (HTRF), immunoprecipitation, western blotting and immunohistochemistry are summarized in Supplementary Table 2. The human HTT exon 2 antibody (AB2644) was generated at Thermo Fisher Scientific and the HTT2a antibodies (BAT-GRK and BAT-TGC) were generated at David’s Biotechnologie GMBH (Regensburg).

### Homogeneous time-resolved fluorescence

HTRF assays were performed as previously described.^10,29,30^ Lysate dilutions and antibody concentrations are summarized in Supplementary Table 3.

### Western blotting and immunoprecipitation

Immunoprecipitations from WT and zQ175 cortical samples were performed using the 3B5H10 antibody (Sigma-Aldrich) as described.^31^ For western blotting, proteins were denatured, separated by 7.5%, 10% or 12% SDS-PAGE (Criterion TGX, Bio-Rad), blotted onto nitrocellulose membranes and detected by chemiluminescence, as described.^31,32^ Primary antibody dilutions were 1:1000.

### Cell culture and transfections

HTT1a and HTT2a expression constructs were generated in pcDNA3.1(+) (Thermo Fisher Scientific) at Azenta Life Sciences. COS-1 cells were transiently transfected with 1.2 μg DNA using Lipofectamine 3000 (Thermo Fisher Scientific), the medium was changed the following day, and cells were harvested after 24 h. Lysates were prepared in HEPES buffer (50 mM HEPES/NaOH (pH 7.0), 150 mM NaCl, 10 mM EDTA, 1.0% Nonidet P40, 0.5% sodium deoxycholate, 0.1% SDS); 20 μg of lysate were analysed by western blot.

### Immunohistochemistry

Mice were transcardially perfusion fixed with 4% paraformaldehyde (Pioneer Research Chemical Ltd.) and tissue processing and the storage of sections was as previously documented.^32^ The immunostaining and imaging of coronal sections was carried out as previously outlined except the ABC reagent was diluted 1:4.^30^ Sections from all ages and genotypes were stained, imaged and processed together. Images were acquired as described.^33^

### Fluorescent resonance energy transfer-based aggregation seeding assay

The fluorescent resonance energy transfer (FRET)-based aggregation assay (FRASE) was performed as previously described, except that 6.15 µg of crude lysate were used per replicate.^32,34^

### Nanopore sequencing

Nanopore long-range sequencing was performed as described.^35^ Reads were aligned against gencode mus musculus version vM29 (GRCm39). Stringtie v2.2.1 was used to generate a consensus gene transcript file across all samples and tissues using the -L and –conservative options.^36^

### RNA sequencing

Total RNA was prepared for 6-month samples and sequenced at Azenta Life Sciences as described.^33^ Twelve-month samples were processed at QBiC (Tübingen) using QIAsymphony RNA extraction kits, the NEBNext Ultra II Directional RNA kits for library preparation and sequenced on a NovaSeq 6000 with paired-end 100 bp reads (S2 Reagent Kit v1.5). Differential expression tests were performed in R using DESeq2, also as described.^33,37^ A posterior probabilities-based method was used to quantify the probability that the intron 1 deletion restored transcriptional dysregulation, either partially or fully, on a gene-by-gene basis.^38^ The dysregulated gene list for *Hdh*Q150 versus wild type was tested for ranked gene set enrichment against the GOBP (gene ontology biological process) version 18.9.29 using the GSEA (gene set enrichment analysis) function within the R clusterProfiler package.^39,40^ The DESeq2 Wald statistic was used to rank genes and a clusterProfiler false discovery rate (FDR) < 0.05 was deemed significant.

### CSF and plasma biomarkers

CSF and plasma biomarker levels were measured as described.^41^

### Statistics

Data were screened for outliers at sample level using the robust regression and outlier removal (ROUT) test (Q = 1%; GraphPad Prism v10) and outliers were removed from the analysis. All data sets were tested for a normal Gaussian distribution (Shapiro–Wilk, Prism v10). Statistical analysis was by one-way ANOVA or two-way ANOVA with Tukey’s *post hoc* tests. Non-parametric analysis was performed by the Kruskal–Wallis test with Dunn’s *post hoc* analysis. Graphs were prepared using Prism v10 (GraphPad Software, California, USA). *P*-values ≤ 0.05 were considered statistically significant.

## Results

The *Htt1a* transcript is generated through the activation of cryptic polyA sites in intron 1 of *Htt* and the termination of transcription. The SoftBerry POLYAH algorithm predicted nine cryptic polyA sites within mouse intron 1 and it is the first two of these sites that are activated in Huntington’s disease knock-in mice.^8,9^ To prevent the production of the HTT1a protein, through the termination of transcription in intron 1, we aimed to delete all potential cryptic polyA sites whilst retaining canonical splicing elements. We chose to use the *Hdh*Q150 knock-in model for this purpose, in which the mouse polyQ encoding sequence: (CAG)_2_CAA(CAG)_4_ had been replaced with an expanded CAG repeat with no other modifications to mouse *Htt.*^42^

A clustered regularly interspaced short palindromic repeat (CRISPR)/Cas9 approach was designed to delete approximately 20 kilobase pairs (kb) from intron 1. In total, 12 founders were obtained with the deletion on the WT allele, and one founder with the deletion on the mutant allele (see Materials and Methods). Colonies were established from a WT founder (WTΔI) and the mutant founder (*Hdh*Q150ΔI). The CAG repeat expansion in the *Hdh*Q150ΔI mice was found to be ∼195, which was well-matched to the CAG repeat size of the *Hdh*Q150 colony.

### The production of the HTT1a protein is much diminished in the *Hdh*Q150ΔI mice

**Figure 1:**
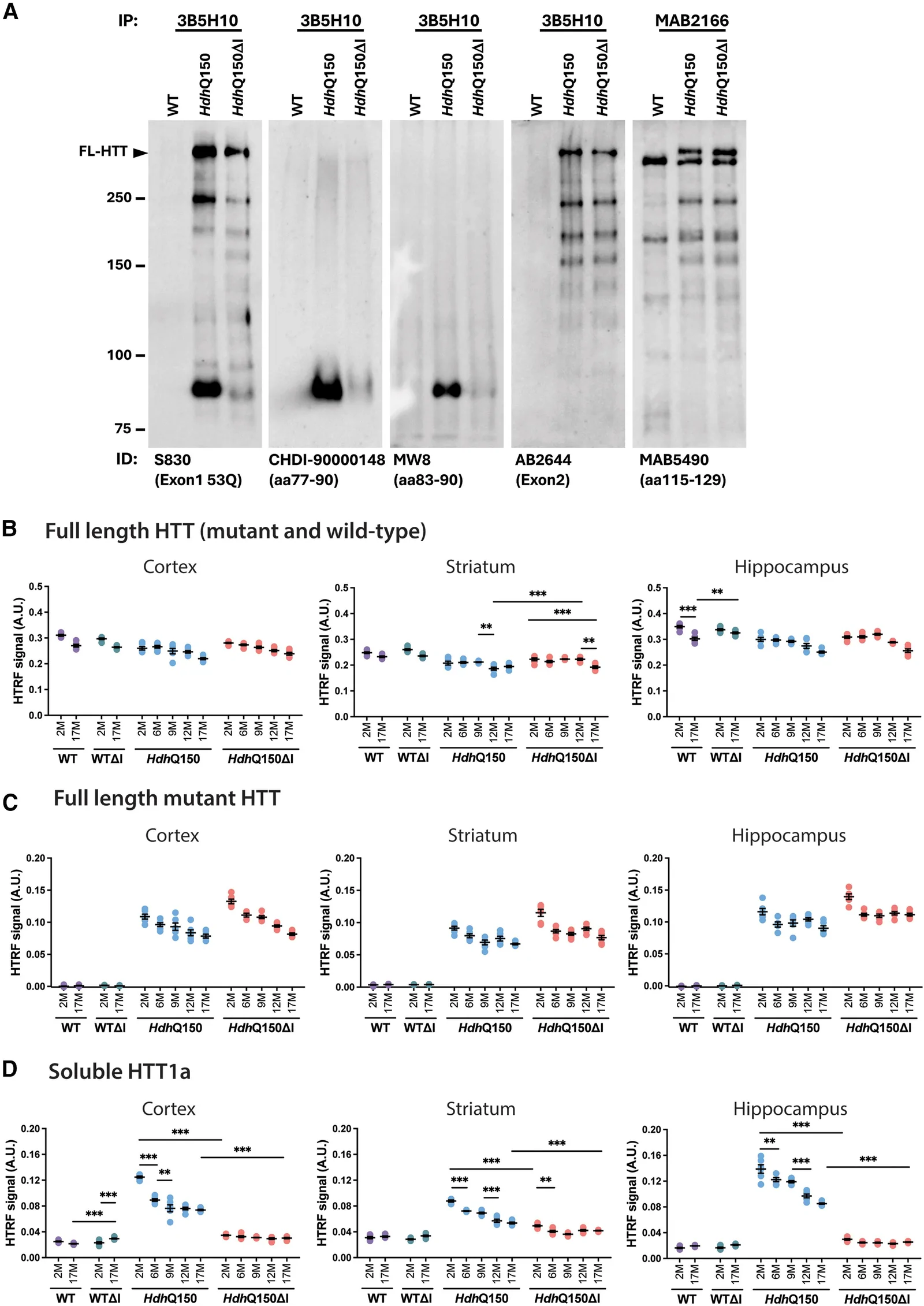
**HTT1a is depleted in *Hdh*Q150ΔI mice.** (**A**) Mutant HTT was immunoprecipitated with 3B5H10 from WT, *Hdh*Q150 and *Hdh*Q150ΔI cortical lysates from mice at 2 months of age and fractionated on a 7.5% SDS-PAGE gel. Western blots were immunoprobed with S830, with the MW8 and CHDI-0148 HTT1a-specific antibodies and with AB2644 that detects exon 2 HTT. Mutant and WT HTT were immunoprecipitated with MAB2166 and the western blot was immunoprobed with MAB5490. Size standards are in kDa. (**B**–**D**) Homogeneous time-resolved fluorescence (HTRF) assays were performed on the cortex, striatum and hippocampus from *Hdh*Q150 and *Hdh*Q150ΔI mice at 2, 6, 9, 12 and 17 months of age and from WT and WTΔI mice at 2 and 17 months. (**B**) Total soluble full-length HTT levels (WT and mutant) were measured using the D7F7-MAB5490 HTRF assay. (**C**) Soluble full-length mutant HTT levels were measured by the MAB5490-MW1 assay. (**D**) Soluble HTT1a protein levels (2B7-MW8 assay) were dramatically decreased in *Hdh*Q150ΔI compared with *Hdh*Q150 brain regions. *n* = 6/genotype, equal sexes. Statistical analysis was two-way ANOVA with Tukey’s *post hoc* correction. Two-way ANOVA comparisons were *Hdh*Q150 versus *Hdh*Q150ΔI and WT versus WTΔI. Error bars show mean ± standard error of the mean. **P ≤ 0.01, ***P ≤ 0.001. aa = amino acids, A.U. = arbitrary units; FL = full-length; ID = immunodetect; IP = immunoprecipitate; M = months; WT = wild-type.

We first investigated whether the HTT1a protein was generated in the *Hdh*Q150ΔI mice. Immunoprecipitation of mutant HTT with the polyQ-specific antibody 3B5H10 was performed on cortical lysates from 2-month-old WT, *Hdh*Q150 and *Hdh*Q150ΔI mice. The immunoprecipitates were immunoprobed with S830, a polyclonal antibody to exon 1 HTT, with MW8 and CHDI-0148 that are neo-epitope antibodies for the C-terminus of the HTT1a protein, and with AB2644 that detects exon 2 HTT (Fig. 1A). As 3B5H10 binds an expanded polyQ tract, it only immunoprecipitates mutant HTT, and therefore, there was no signal in the WT lanes. S830 detected full-length HTT and the characteristic pattern of HTT fragments.^31^ Although HTT1a is the smallest HTT fragment, it does not migrate fastest through the gel.^31^ The band representing HTT1a had a much lower intensity in the *Hdh*Q150ΔI samples (Fig. 1A); that this band represented HTT1a was confirmed by immunoprobing with the HTT1a-specific antibodies CHDI-0148 and MW8, and by its absence when probing with AB2644, an antibody to exon 2 HTT (Fig. 1A). The cortical lysates were also immunoprecipitated with MAB2166 (443–457 amino acids) and immunoprobed with MAB5490 (115–129 amino acids). This precipitated both WT and mutant proteins, confirming the genotypes of the mice. Overall, the relative intensities of full length HTT and its proteolytic fragments were comparable between *Hdh*Q150 and *Hdh*Q150ΔI mice, and the corresponding HTT fragments migrated much faster in the WT sample (Fig. 1A).

We applied HTRF bioassays to compare the levels of the HTT and HTT1a proteins in the cortex, striatum and hippocampus of WT, WTΔI, *Hdh*Q150 and *Hdh*Q150ΔI mice and to determine how these changed with age.^10^  ^,29^ Samples were prepared from brain regions at 2, 6, 9, 12 and 17 months of age for the *Hdh*Q150 and *Hdh*Q150ΔI mice and at 2 and 17 months for WT and WTΔI mice (Fig. 1B–D). Overall, total soluble full-length HTT levels (mutant and WT: D7F7—MAB5490) were comparable between WT and WTΔI mice and between *Hdh*Q150 and *Hdh*Q150ΔI mice over the 17-month period (Fig. 1B). Similarly, there was no apparent difference in the levels of soluble full-length mutant HTT levels (MAB5490—MW1) between *Hdh*Q150 and *Hdh*Q150ΔI mice (Fig. 1C). In contrast, the levels of the soluble HTT1a protein decreased with age in the *Hdh*Q150 brain regions, due to recruitment into HTT aggregates, and HTT1a was barely detectable in the *Hdh*Q150ΔI brain regions by the 2B7-MW8 HTRF assay (Fig. 1D).

### HTT aggregation appears much later in the brains of *Hdh*Q150ΔI, as compared with *Hdh*Q150 mice

**Figure 2:**
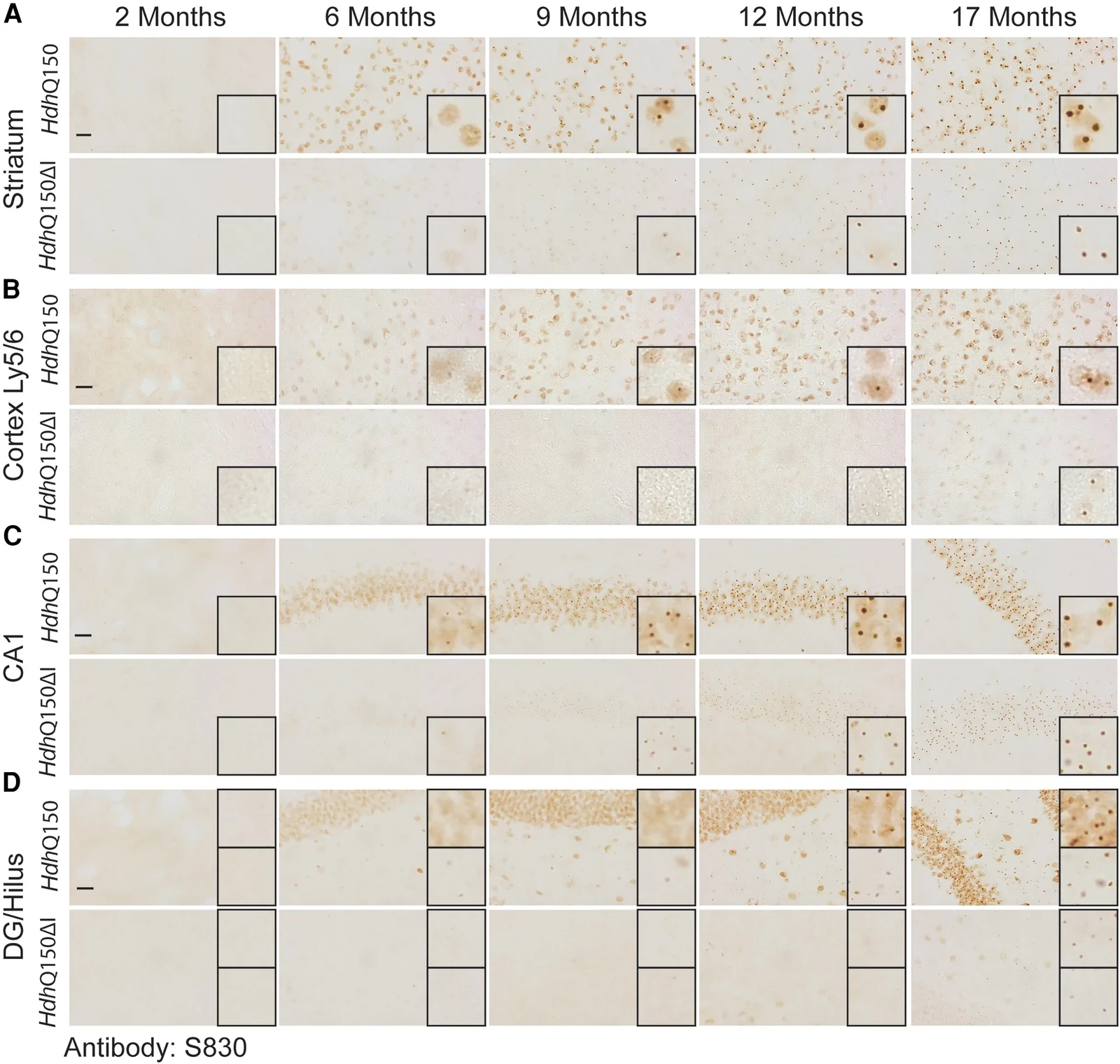
**HTT aggregation appears much later in the brains of *Hdh*Q150ΔI mice.** Coronal sections were immunostained with the S830 antibody and imaged for (**A**) striatum, (**B**) layers 5/6 of the cortex, (**C**) CA1 of the hippocampus and (**D**) dentate gyrus and hilus of the hippocampus for *Hdh*Q150 and *Hdh*Q150ΔI animals at 2, 6, 9, 12 and 17 months of age. In zoomed images, nuclear aggregation is on the *bottom* in **A**, **B** and **C** and on the *top* for the dentate gyrus in **D**. Neuropil aggregation in the hilus is on the bottom in (**D**). *n* = 3. Scale bar = 20 μm, zoomed images are 20 μm^2^. Ly = layers; DG = dentate gyrus.

Brains from *Hdh*Q150 and *Hdh*Q150ΔI mice at 2, 6, 9, 12 and 17 months of age and from WT and WTΔI mice at 17 months of age were sectioned and immunostained with the S830 antibody. S830 is a polyclonal antibody that was raised against a mutant version of human exon 1 HTT. Although some of its epitopes are specific to human HTT, some cross-react with mouse HTT. By 6 months of age, nuclear HTT aggregation was apparent in the striatum, cortex (layers 5/6), CA1 and dentate gyrus regions of the hippocampus in the form of diffuse nuclear aggregation (Fig. 2A–D). Nuclear inclusions in the form of small puncta were also apparent in some nuclei. As the mice aged, nuclear inclusions could be identified in all aggregation-containing nuclei and the diffuse aggregation became less intense (Fig. 2A–D). Extranuclear inclusions were also apparent in the hilus at 9 months and increased with age (Fig. 2D). There was no signal on WT or WTΔI sections immunostained with S830 (Supplementary Fig. 1).

This aggregation process was considerably delayed in the *Hdh*Q150ΔI mice. Inclusions were not apparent in the striatum and CA1 regions of the hippocampus until 9 months of age (Fig. 2A and C), and in layer 5/6 of the cortex and the dentate gyrus until 17 months (Fig. 2B and D). HTT aggregation was apparent as small inclusions, and the diffuse nuclear aggregation was largely absent (Fig. 2A–D).

We applied the FRET-based aggregation seeding assay (FRASE)^34^ to determine when aggregate seeds could first be identified in these brain regions by this approach. At 6 months of age, a FRASE signal could be detected in the striatum of *Hdh*Q150 mice, but not from *Hdh*Q150ΔI mice (Supplementary Fig. 2). At 6 months, seeds could not be detected by the FRASE assay in the cortex or hippocampus of *Hdh*Q150 mice (Supplementary Fig. 2).

### HTT inclusions in the *Hdh*Q150ΔI mice contained HTT1a

**Figure 3:**
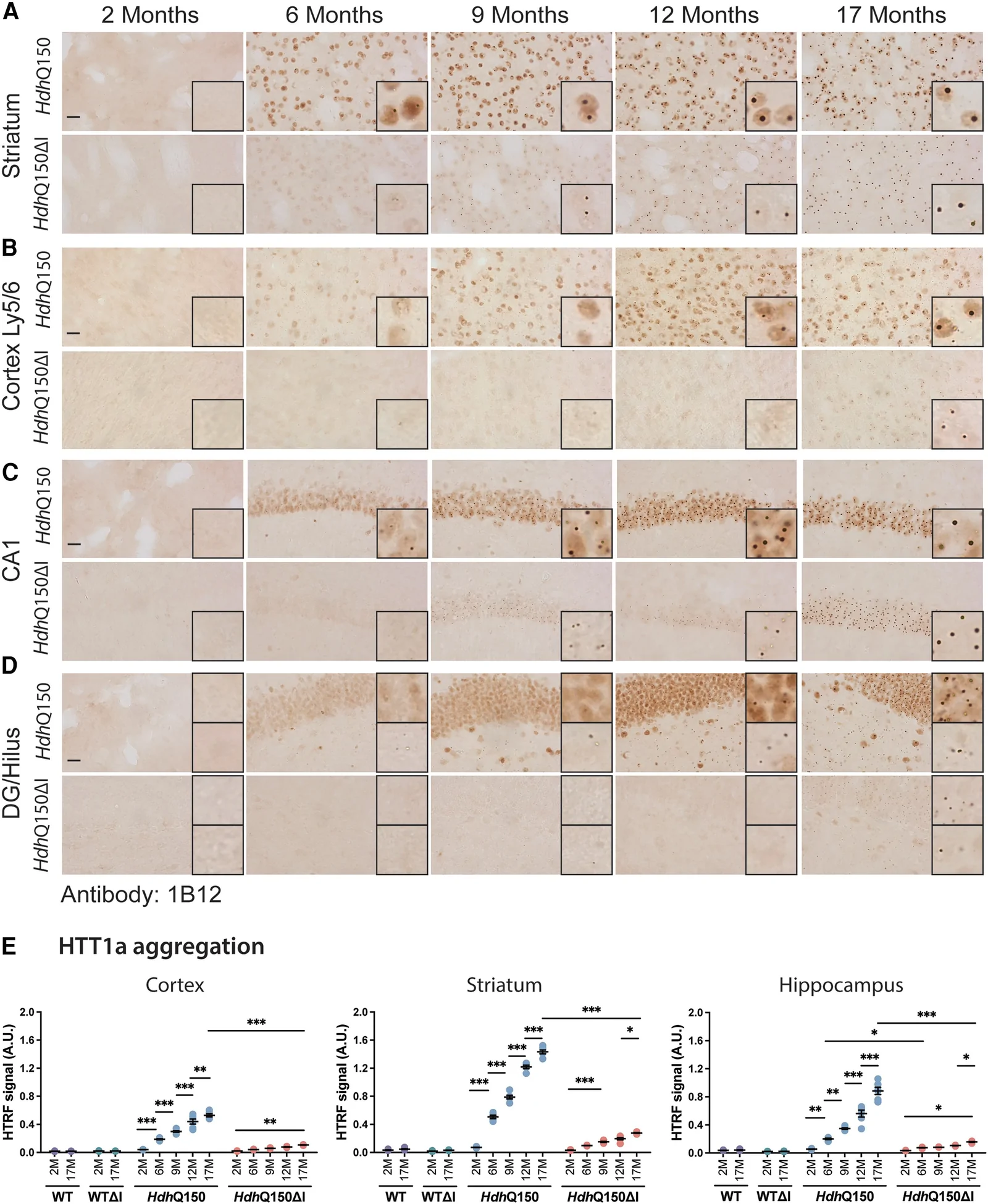
**HTT1a is present in the HTT aggregates in the *Hdh*Q150ΔI mice.** Coronal sections of the brain were immunostained with the 1B12 antibody and imaged for (**A**) striatum, (**B**) layers 5/6 of the cortex, (**C**) CA1 of the hippocampus and (**D**) dentate gyrus and hilus of the hippocampus for *Hdh*Q150 and *Hdh*Q150ΔI animals at 2, 6, 9, 12 and 17 months of age. In zoomed images, nuclear aggregation is on the *bottom* in **A**, **B** and **C** and on the *top* for the dentate gyrus in **D**. Neuropil aggregation in the hilus is on the *bottom* in **D**. *n* = 3. Scale bar = 20 μm, zoomed images are 20 μm^2^. Ly = layers; DG = dentate gyrus; WT = wild type. (**E**) Aggregated HTT1a levels were measured by homogeneous time-resolved fluorescence (HTRF) (MW8—CHDI-1414 assay) in the cortex, striatum and hippocampus from *Hdh*Q150 and *Hdh*Q150ΔI mice at 2, 6, 9, 12 and 17 months of age and from WT and WTΔI mice at 2 and 17 months. *n* = 6/genotype, equal sexes. Statistical analysis was two-way ANOVA with Tukey’s *post hoc* correction. Two-way ANOVA comparisons were *Hdh*Q150 versus *Hdh*Q150ΔI and WT versus WTΔI. Error bars show mean ± standard error of the mean. *P ≤ 0.05, **P ≤ 0.01, **P ≤ 0.001. A.U. = arbitrary units; M = months; WT = wild type.

The sections from *Hdh*Q150 and *Hdh*Q150ΔI mice at 2, 6, 9, 12 and 17 months of age and from WT and WTΔI mice at 17 months of age were also immunostained with the HTT1a-specific 1B12 antibody. Immunostaining with 1B12 was stronger than that produced by S830, but the timing of the appearance of HTT aggregates in the *Hdh*Q150 brain regions, in the form of diffuse aggregation or inclusions, was the same as detected by S830 (Fig. 3A–D). Immunostaining of *Hdh*Q150ΔI sections with 1B12 indicated that they contained aggregated HTT1a (Fig. 3). As before, in the *Hdh*Q150ΔI sections, nuclear inclusions were not seen in the striatum or CA1 region of the hippocampus until 9 months (Fig. 3A and C), but a diffuse nuclear stain was apparent in the striatum from 6 months, that could also be seen in the S830 stained striatal sections on closer inspection (Figs 2A and 3A). 1B12 did not identify HTT1a aggregation in layers 5/6 of the cortex or the dentate gyrus/hilus until 17 months as with S830 (Fig. 3B and D). There was no signal on WT or WTΔI sections immunostained with 1B12 (Supplementary Fig. 1).

To confirm that HTT1a was present in HTT aggregates in the *Hdh*Q150ΔI brains and to compare levels with *Hdh*Q150 mice, we employed HTRF assays on brain region lysates. HTRF and meso scale discovery (MSD) assays that utilize the 4C9 and MW8 antibodies are widely used to assess aggregated HTT levels,^10,29,43,44^ and these assays have been shown to be specific for aggregated HTT1a.^29,30^ However, the 4C9 antibody was raised against the polyproline-rich region in human HTT and does not recognize mouse HTT.^44^ To generate assays that detect aggregated mouse HTT1a, the CHDI-1414 antibody that is specific to the mouse polyproline region was substituted for 4C9.^30^

The MW8-CHDI-1414 HTRF aggregation assay was performed on the same lysates and in parallel to the soluble HTT and HTT1a assays (Fig. 1B–D). These were from the cortex, striatum and hippocampus at 2, 6, 9, 12 and 17 months of age for the *Hdh*Q150 and *Hdh*Q150ΔI mice and at 2 and 17 months of age for WT and WTΔI mice. HTT1a aggregation could be detected in all three *Hdh*Q150 brain regions by 6 months and increased with age to 17 months (Fig. 3E). The level of HTT1a aggregation in the *Hdh*Q150ΔI brain regions was much lower, where statistically significant levels of aggregation were present in the striatum at 9 months of age and not until 17 months in the cortex and hippocampus (Fig. 3E), consistent with immunohistochemistry data.

### RNA transcripts generated from the *Htt* loci in *Hdh*Q150, *Hdh*Q150ΔI, WT and WTΔI brain regions

**Figure 4:**
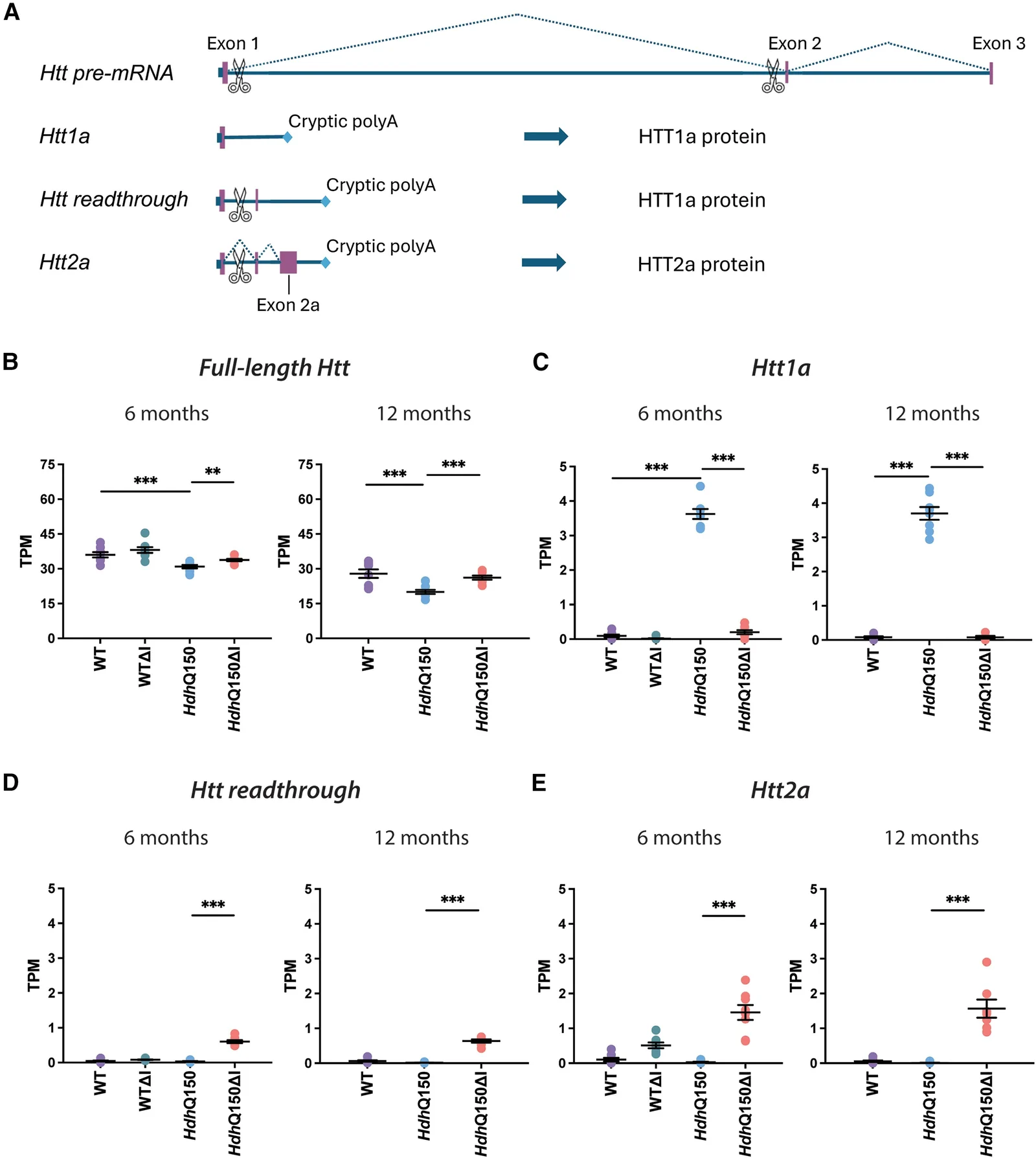
**Striatal huntingtin transcripts generated from the genetically modified loci.** (**A**) Schematic illustrating the 5′ end of the *Htt* gene and the transcripts identified by nanopore sequencing. *Htt1a* contains exon 1 and intron 1 sequences and is translated to produce the HTT1a protein. *Htt readthrough* contains exon 1, the deleted intron 1, exon 2 and intron 2 sequences; if translated it would generate the HTT1a protein. *Htt2a* contains exon 1, exon 2, and a novel exon 2a located in intron 2 and intron 2 sequences; if translated it would generate the HTT2a protein (Supplementary Fig. 4A). (**B**–**E**) Quantification of (**B**) the full length *Htt* transcript, (**C**) the *Htt1a* transcript, (**D**) the *Htt readthrough* transcript and (**E**) the *Htt2a* transcript in WT, WTΔI, *Hdh*Q150 and *Hdh*Q150ΔI striatum at 6 months of age and WT, *Hdh*Q150 and *Hdh*Q150ΔI striatum at 12 months. *n* = 8/genotype, equal sexes. Statistical analysis was by DESeq2 with Benjamini–Hochberg correction. Error bars show mean ± standard error of the mean. **P ≤ 0.01, ***P ≤ 0.001. TPM (transcripts per million) were computed from RNA sequencing data at the indicated ages. WT = wild type.

We next performed nanopore sequencing to define the *Htt* mRNA isoforms that were transcribed from the *Hdh*Q150, *Hdh*Q150ΔI, WT and WTΔI loci. The *Hdh*Q150, *Hdh*Q150ΔI and WTΔI mice were bred to homozygosity and striatum, cortex and hippocampus were harvested at 2 months of age, along with these brain regions from WT mice. Nanopore sequencing was performed (*n* = 5–6/genotype/brain region) and four CAG repeat-containing isoforms were identified: the canonically spliced transcript (*Htt*), *Htt1a*, the *Htt readthrough* and *Htt2a* (Fig. 4A). The *Htt readthrough* transcript contained *Htt* exon 1, the deleted intron 1, exon 2 and terminated at the first cryptic polyA site in intron 2; if translated this would generate the HTT1a protein (Fig. 4A). The *Htt2a* transcript contained *Htt* exon 1, exon 2 and a novel exon 2a located in intron 2 terminating at the first cryptic polyA site in intron 2; if translated this would generate the HTT2a protein (Fig. 4A).

Illumina-based RNA sequencing was performed on the striatum and hippocampus from WT, WTΔI, *Hdh*Q150 and *Hdh*Q150ΔI mice at 6 months of age and from WT, *Hdh*Q150 and *Hdh*Q150ΔI at 12 months, and the level of each of the four transcripts: *Htt*, *Htt1a*, *Htt readthrough* and *Htt2a* was quantified (Fig. 4B–E and Supplementary Fig. 3). The spliced full-length *Htt* transcript was present in all genotypes as would be expected. The level in *Hdh*Q150 striatum and hippocampus was lower than that in WT mice at both 6 and 12 months, whereas full-length *Htt* levels in these *Hdh*Q150ΔI brain regions did not differ from WT (Fig. 4B and Supplementary Fig. 3A). The *Htt1a* transcript was present in *Hdh*Q150, and not WT, striatum and hippocampus as expected (Fig. 4C and Supplementary Fig. 3B). *Htt1a* was absent from these brain regions in the *Hdh*Q150ΔI mice, indicating that the removal of the cryptic polyA sites from intron 1 had prevented transcription termination in intron 1 as predicted. The *Htt readthrough* product was present in the *Hdh*Q150ΔI striatum and hippocampus at both 6 and 12 months of age (Fig. 4D and Supplementary Fig. 3C). Translation of the *Htt readthrough* transcript would generate the HTT1a protein, indicating that this small transcript is most likely translocated to the cytoplasm and is the origin of the low levels of HTT1a detected in the *Hdh*Q150ΔI mice. The *Htt2a* transcript could also be detected in the striatum and hippocampus of WTΔI and *Hdh*Q150ΔI mice, with greater levels in *Hdh*Q150ΔI (Fig. 4E and Supplementary Fig. 3D). Two antibodies were raised against HTT2a (BAT-GRK and BAT-TGC) (Supplementary Fig. 4A). These detected the HTT2a protein in lysates from COS-1 cells transiently transfected with a *Htt2a* construct (Supplementary Fig. 4B) but failed to detect HTT2a in brain lysates from *Hdh*Q150ΔI mice (Supplementary Fig. 4C).

### Transcriptional dysregulation is delayed in *Hdh*Q150ΔI brain regions

Transcriptional dysregulation has been extensively studied in mouse models of Huntington’s disease.^45^ Therefore, the striatal and hippocampal data sets from WT, WTΔI, *Hdh*Q150 and *Hdh*Q150ΔI mice at 6 months of age, and WT, *Hdh*Q150 and *Hdh*Q150 mice at 12 months were used to determine the effect of decreasing HTT1a levels, and delaying the deposition of nuclear HTT aggregation, on transcriptional dysregulation.

**Figure 5:**
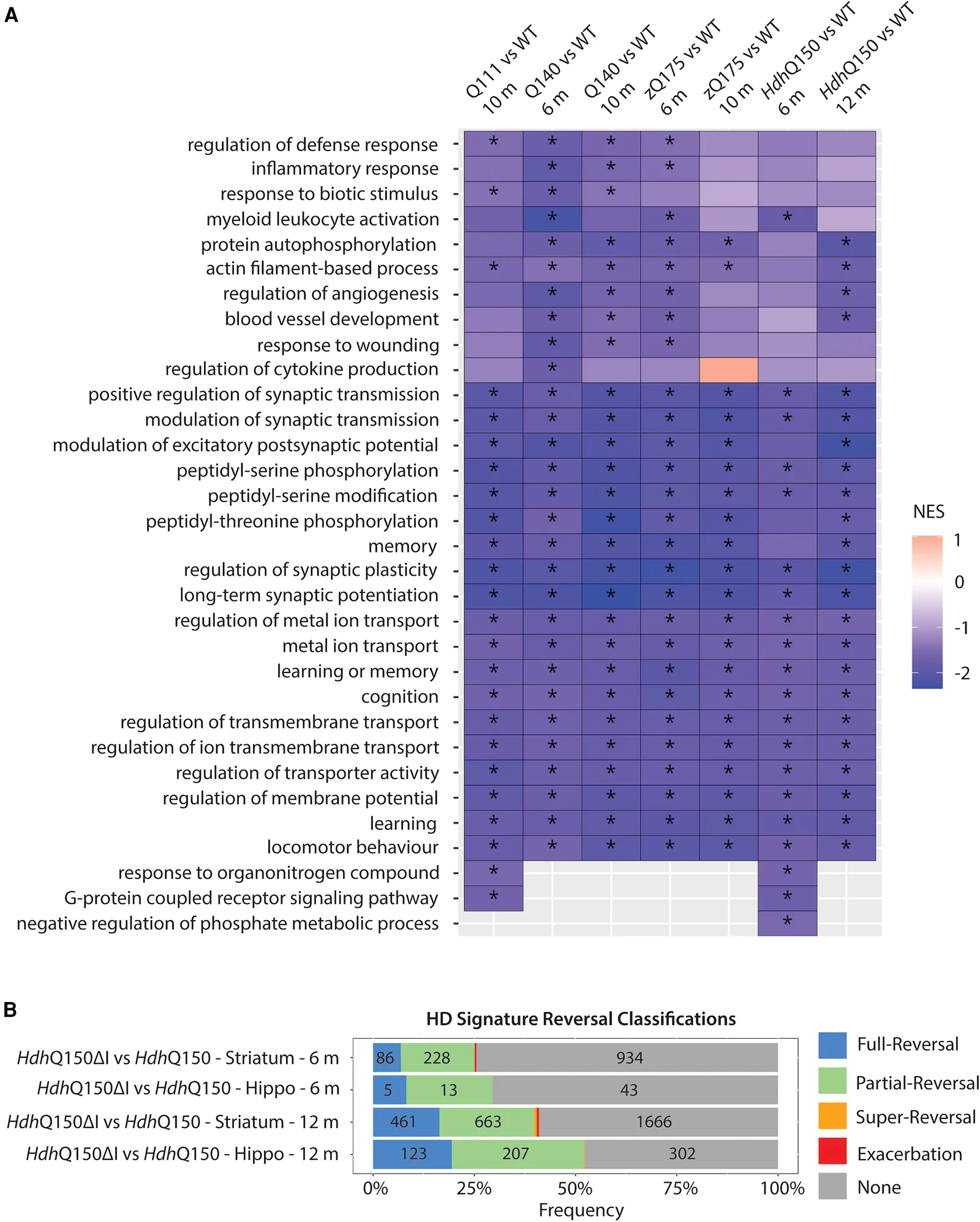
**The reduction in the HTT1a protein improves transcriptional dysregulation in the striatum and hippocampus of *Hdh*Q150ΔI mice.** (**A**) A common signature of HD-related biological processes dysregulation across different mouse models. The differentially expressed genes (RNA-seq) in the striatum of *Hdh*Q111 mice at 10 months of age, Q140 mice at 6 and 10 months, zQ175 mice at 6 and 10 months, and *Hdh*Q150 mice at 6 and 12 months were analysed by ranked gene set enrichment analysis (GSEA) against the gene ontology biological process (GOBP) collection. NES = normalized enrichment score; m = months. The star indicates that the dysregulated process has an *FDR* < 0.05 (false discovery rate). (**B**) Reversal of the Huntington's disease signature in *Hdh*Q150ΔI mice. The proportion of dysregulated genes for which the level of expression was reversed towards WT levels in the *Hdh*Q150ΔI striatum and hippocampus at 6 months of age and the *Hdh*Q150ΔI striatum and hippocampus at 12 months compared with *Hdh*Q150 mice. *n* = 8/genotype, equal sexes. Hippo = hippocampus.

We began by comparing the profile of dysregulated genes in the striatum of *Hdh*Q150 mice at 6 and 12 months with that in other knock-in mouse models of Huntington’s disease, using archival striatal RNA sequencing data for *Hdh*Q111 mice at 10 months of age, Q140 mice at 6 and 10 months, and zQ175 mice at 6 and 10 months.^45^ The differential gene expression results for each of these data sets were subjected to ranked gene set enrichment analysis (GSEA) against the gene ontology biological process (GOBP) collection to identify biological pathways that were significantly dysregulated in the striatum of each of the models [false discovery rate (*FDR*) < 0.05]. The profiles were very similar with 16 biological processes dysregulated in all knock-in models at all ages (Fig. 5A). The dysregulated *Hdh*Q150 striatal profile at 6 months of age most closely aligned with that from *Hdh*Q111 striatum at 10 months. All dysregulated biological processes in the *Hdh*Q150 striatum at 12 months of age were also dysregulated in the Q140 and zQ175 striata (Fig. 5A).

The impact of the *Htt2a* transcript on the transcriptome in the WT striatum was assessed by comparing the RNA sequencing data sets of WTΔI and WT striata at 6 months of age. Differential gene expression analysis identified 15 genes for which the expression levels were altered (Supplementary Table 4). The level of *Htt2a* expression was higher in the *Hdh*Q150ΔI striatum than in the WTΔI striatum (Fig. 4E). However, it was not possible to assess the effect of *Htt2a* on the *Hdh*Q150 striatal transcriptome, because of the large number of genes (>1000) that were dysregulated due to the Huntington’s disease mutation (Fig. 5A and Supplementary Table 5). Given that only 15 genes were dysregulated in the WTΔI striata, any contribution from the *Htt2a* transcript to the Huntington’s disease dysregulated signature is likely to be small.

Next, we determined the effect of decreasing HTT1a levels on the *Hdh*Q150 dysregulated gene signature at 6 months of age. Of the 1254 dysregulated genes in the *Hdh*Q150 striata, the dysregulation was improved for 25% of the genes (86 fully reversed and 228 partially reversed) in the *Hdh*Q150ΔI mice (Fig. 5B and Supplementary Table 5). At 6 months of age, only 61 genes were dysregulated in the hippocampus, which was too few to draw any strong conclusions, but there was a similar pattern to the striatum in that for 30% of them, the dysregulation was either fully or partially ‘reversed’ in the *Hdh*Q150ΔI hippocampus (Fig. 5B and Supplementary Table 5). At 12 months of age, there were 2820 dysregulated genes in the *Hdh*Q150 striatum, and 697 dysregulated genes in the *Hdh*Q150 hippocampus. Of these, the dysregulated expression level of 40% in the striatum and 52% in the hippocampus were either fully or partially reversed in the *Hdh*Q150ΔI mice (Fig. 5B and Supplementary Table 5).

### CSF and plasma biomarkers are maintained at wild-type levels in the *Hdh*Q150ΔI mice

**Figure 6:**
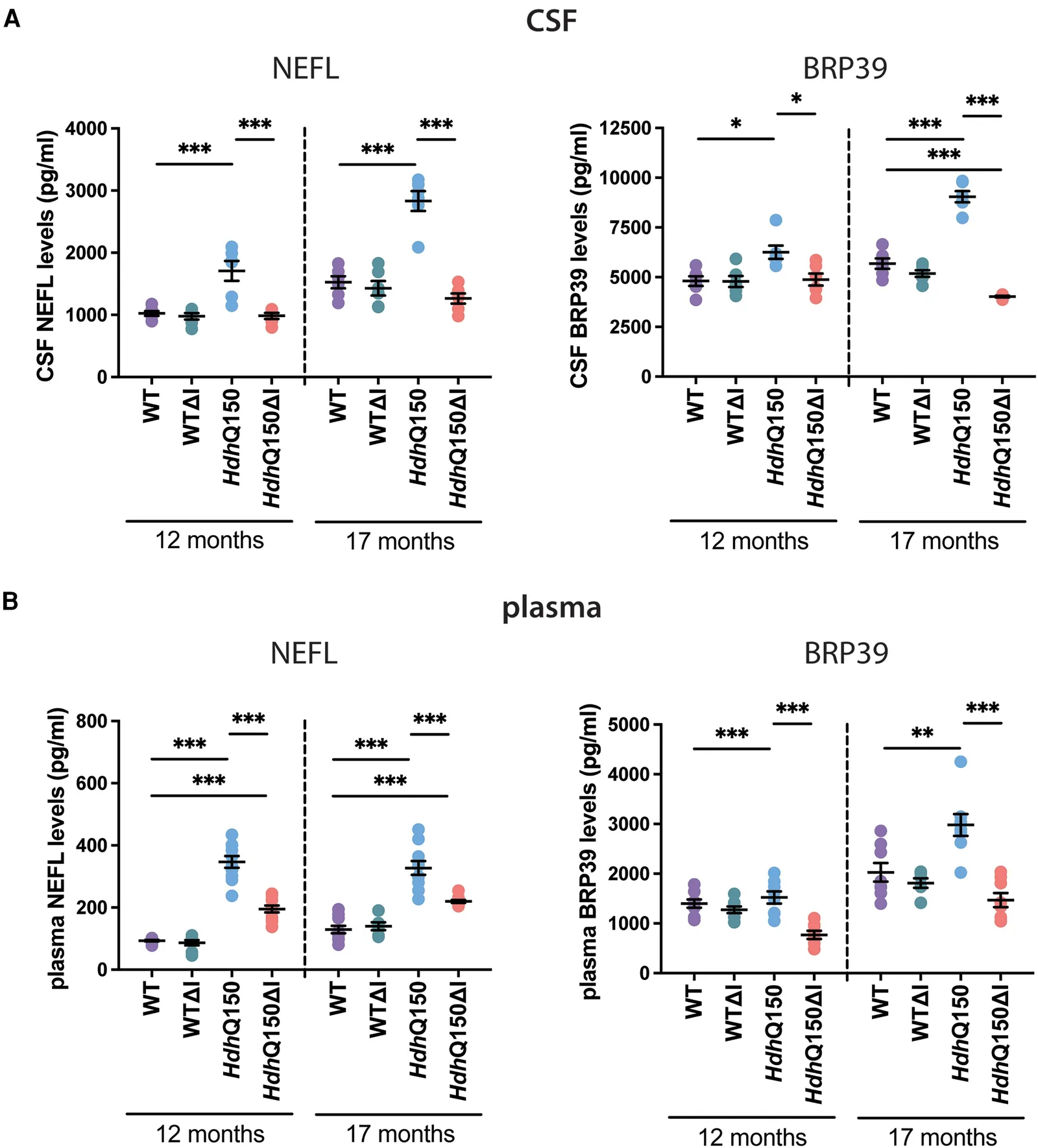
**Plasma and CSF biomarkers are at wild-type levels in *Hdh*Q150ΔI mice.** Neurofilament light polypeptide (NEFL) and BRP39 were measured in WT, WTΔI, *Hdh*Q150 and *Hdh*Q150ΔI CSF and plasma at 12 and 17 months of age. (**A**) NEFL and BRP39 levels were raised in *Hdh*Q150 CSF by 12 months of age but remained at WT levels in *Hdh*Q150ΔI CSF even at 17 months. (**B**) NEFL and BRP39 levels were raised in *Hdh*Q150 plasma by 12 months of age. The increase in plasma NEFL was much reduced in *Hdh*Q150ΔI mice and BRP39 remained at WT levels in *Hdh*Q150ΔI plasma even at 17 months of age. *n* = 6–10/genotype, 3–5/sex. Statistical analysis was one-way ANOVA with Tukey’s *post hoc* correction or Kruskal–Wallis with Dunn’s *post hoc* correction. Error bars show mean ± standard error of the mean. *P ≤ 0.05, **P ≤ 0.01, ***P ≤ 0.001. WT = wild type.

Plasma and CSF biomarkers provide a read-out that is directly translatable to Huntington’s disease clinical measures. Neurofilament light polypeptide (NEFL) (alias NfL)^46-48^ and YKL40^48,49^ have been extensively studied in Huntington’s disease CSF and plasma and both NEFL and BRP39 (mouse YKL40) can be robustly detected in the CSF and plasma of mouse models of Huntington’s disease.^41^ We measured the levels of NEFL and BRP39 in these biofluids from *Hdh*Q150, *Hdh*Q150ΔI, WT and WTΔI mice (Fig. 6). NEFL and BRP39 levels were raised in *Hdh*Q150 CSF by 12 months of age but remained at WT levels in the *Hdh*Q150ΔI mice at both 12 and 17 months (Fig. 6A). The levels of NEFL and BRP39 were lower in plasma than in CSF (Fig. 6B). In plasma, NEFL was raised compared with WT mice in both *Hdh*Q150 and *Hdh*Q150ΔI mice by 12 months, but the extent to which this had occurred was significantly lower in the *Hdh*Q150ΔI mice (Fig. 6B). An increase in plasma BRP39 was also detected by 12 months of age in *Hdh*Q150 mice but was at WT levels in *Hdh*Q150ΔI mice at both 12 and 17 months (Fig. 6B). Overall, NEFL and BRP39 in WTΔI mice were at WT levels in CSF and plasma at both 12 and 17 months of age (Fig. 6).

## Discussion

Somatic CAG repeat expansion causes the progressive elongation of the CAG repeat mutation in the brains of people with Huntington’s disease with age. Single-cell nuclear sequencing studies have proposed that the mutation remains benign until the repeat has expanded beyond a critical threshold of more than (CAG)_100_.^17^ Given that the level of the pathogenic HTT1a protein increases with increasing CAG length, could this be the effector through which somatic CAG expansion exerts its detrimental effects? We have applied a genetic strategy to prevent the production of HTT1a in a knock-in mouse model of Huntington’s disease that resulted in only very low levels being produced. This had the consequence of delaying HTT aggregation in the brain by months, improving transcriptional dysregulation and competingly retaining CSF biomarkers at WT levels. These data support the hypothesis that HTT1a contributes significantly to Huntington’s disease pathogenesis.

The *Hdh*Q150 knock-in model was chosen for this study because the strategy used for its creation replaced the mouse (CAG)_2_CAA(CAG)_4_ sequence with an expanded CAG repeat and did not result in any other modifications to the mouse *Htt* sequence.^42^ This is in contrast to other knock-in models, e.g. *Hdh*Q111, Q140, zQ175, in which the replacement of mouse exon 1 *Htt* with human exon 1 *HTT* led to alterations within the 5′ region of intron 1.^50,51^ The deletion of all potential cryptic polyA sites from intron 1 prevented termination of the transcription in the intron as intended, completely preventing the production of the *Htt1a* transcript in *Hdh*Q150ΔI mice. However, in some cases, the deleted intron 1 in the *Hdh*Q150ΔI mice was not spliced and the shortened intron 1 led to the activation of the first cryptic polyA site in intron 2 (*Htt readthrough* transcript). The presence of low levels of the HTT1a protein in *Hdh*Q150ΔI mice is most likely due to the translation of these *Htt readthrough* transcripts. The deleted intron also resulted in the production of the *Htt2a* transcript, which was present in both WTΔI and *Hdh*Q150ΔI mice and, therefore, its generation was not dependent on the presence of an expanded CAG repeat, although levels were higher in the *Hdh*Q150ΔI mice. We failed to detect any HTT2a protein in *Hdh*Q150ΔI brain lysates and so if *Htt2a* is translated, it is at very low levels.

The detection of the HTT1a protein is not straightforward; it does not contain any amino acids that are not present in full-length HTT, and therefore, its detection depends on the use of neo-epitope, conformation-specific antibodies to the C-terminus of HTT1a. The MW8 antibody has fulfilled this role and specifically detects soluble HTT1a on western blots of immunoprecipitated protein,^8,31^ and in HTRF, MSD and AlphaLISA assays for soluble and aggregated HTT1a.^29,30^ The use of MW8 against western blots of immunoprecipitated mutant HTT showed that HTT1a was present at only very low levels in the *Hdh*Q150ΔI brain. But MW8 is a weak antibody, and the HTT1a-specific HTRF assay (2B7-MW8) did not detect such low HTT1a levels. More sensitive HTT1a-specific antibodies have been developed by the CHDI Foundation (e.g. 1B12),^52^ and whilst we were able to use 1B12 for immunohistochemistry, it was not available in time for the bioassay work.

The very low levels of soluble HTT1a in the *Hdh*Q150ΔI brain delayed the appearance of aggregated HTT in the form of diffuse nuclear aggregation and inclusion bodies by several months. Immunohistochemistry with the 1B12 antibody demonstrated that the aggregates were composed of HTT1a in both *Hdh*Q150 and *Hdh*Q150ΔI brains. Whether HTT aggregates contain HTT proteins other than HTT1a is not certain. We have never been able to detect aggregated HTT in knock-in mouse brains with antibodies that detect epitopes C-terminal to HTT1a.^30^ However, we previously showed that, as HTT aggregated, the soluble levels of the two smallest proteolytic fragments of HTT, as well as HTT1a, decreased with age in the *Hdh*Q150 brain, suggesting that all three contributed to the threshold concentration required for HTT aggregation.^31^ Our novel MSD assays also suggested that HTT fragments longer than HTT1a were present in HTT aggregates at low levels, which would be consistent with at least a low level of recruitment of longer HTT fragments.^29^

The deposition of aggregated HTT in the brain and transcriptional dysregulation, especially in the striatum, are molecular phenotypes that appear early in the disease course in all knock-in models. That they have become standard read-outs in preclinical studies for HTT-lowering studies is valid,^33,38,53^ as they are proximal to the mutation, and are phenotypes that are present in the human Huntington’s disease brain.^54-56^ Transcriptional dysregulation in the *Hdh*Q150 striatum was found to have a comparable signature to that in other knock-in mouse models of Huntington’s disease and was consistent with phenotypes in the *Hdh*Q150 line developing at a slower rate than knock-in lines with a mutated version of human exon 1 *HTT*, even with comparable CAG repeat length mutations.^57^ The transcriptional dysregulation in the *Hdh*Q150ΔI striatum was delayed, such that transcription levels were improved in 25% of genes at 6 months of age and 40% at 12 months of age. Given the low level of HTT1a in *Hdh*Q150ΔI mice, a greater improvement might have been expected. However, immunohistochemistry with the 1B12 antibody revealed a faint diffuse aggregated HTT1a signal in striatal nuclei at 6 months of age, that was absent at 2 months. Therefore, there was sufficient HTT1a, probably together with small HTT proteolytic fragments, to initiate the aggregation process, resulting in some transcriptional dysregulation. However, the subsequent aggregate polymerization was substantially delayed. These data are comparable to several HTT-lowering preclinical studies, in which the ‘reversal’ of gene dysregulation was consistently less than 50%.^38,58^

The choice of functional read-outs for preclinical studies in all knock-in mouse models of Huntington’s disease is limited. They do not develop robust behavioural phenotypes that are reproducible between laboratories, have good statistical power or develop at ages amenable to the design of preclinical trials.^59,60^ However, CSF and plasma biomarkers such as NEFL and BRP39 (YKL40 in humans) can be detected in Huntington’s disease mouse biofluids,^41^ and have the advantage that they are directly translatable to clinical trials.^47,49^ We found that NEFL and BRP39 were at WT levels in the *Hdh*Q150ΔI CSF indicating that the reduction in HTT1a in the brain had prevented the disease-associated increase in these biomarkers. This complete rescue of the CSF biomarkers had occurred despite the fact that more than 50% of the Huntington’s disease transcriptional signature remained dysregulated.

Somatic CAG repeat expansion drives the age of onset of Huntington’s disease and the allelic series of knock-in mouse models has been used to determine the effect of a repeat expansion from (CAG)_50_ to (CAG)_190_ on the levels of huntingtin transcripts and protein isoforms.^10^ As the CAG repeat expanded, *Htt1a* mRNA and HTT1a protein levels increased, whilst full-length mutant HTT levels decreased.^10^ Single-cell nuclear sequencing from post-mortem brains has shown that, in the caudate-putamen, CAG repeat expansion is specific to spiny projection neurons (medium spiny neurons).^16,17^ Expansion from an inherited allele of (CAG)_40–43_ had occurred in most neurons to a median length of (CAG)_60–73_ with 2%–5% cells having highly expanded alleles of (CAG)_100 to >500._^17^ That only a small proportion of alleles have such long repeat tracts explains why the *HTT1a* transcripts were easier to detect in brains from juvenile onset cases with inherited alleles of (CAG)_˃90_. In these juvenile cases, as in the *Hdh*Q111, Q140 and zQ175 mouse lines, somatic expansion starts from a highly expanded baseline compared with adult-onset Huntington’s disease brains.^9,61^ For the same reason, the detection of the HTT1a protein in CSF for target engagement in clinical trials is likely to be challenging, even with the more potent 1B12 HTT1a-specific antibodies and platforms with greater sensitivity.^52,62^

There are several HTT-lowering modalities that have progressed to clinical evaluation. Agents that only target full-length *HTT* (mutant and wild type) include the antisense oligonucleotide (ASO) tominersen^63^ and the small molecule splicing modulators branaplam^64^ and PTC518.^65^ A large phase 3 trial of tominersen was halted prematurely because it showed no benefit, and for safety reasons.^66^ Agents that target exon 1 *HTT* sequences lower both full-length HTT and HTT1a and include AMT130, a micro RNA packaged in an adeno-associated virus,^67,68^ ALN-HTT02, a small interfering RNA that targets exon 1^69^ and V0659, an ASO that targets CAG repeats.^65^ Recent preclinical studies using siRNAs and ASOs have highlighted the importance of decreasing HTT1a, showing that this is much more beneficial than lowering full-length HTT alone.^58,70^ The evaluation of patients 36 months after treatment with the AMT-130 gene therapy has also supported this conclusion, with a 75% slowing of disease progression and a reduction in CSF NEFL from baseline in those who had received the high dose (www.uniqure.com/investors-media/press-releases). Given the safety concerns of lowering WT HTT,^71,72^ a therapy that targeted HTT1a alone would have advantages and could be achieved with siRNAs against intron 1 *HTT* sequences. The data presented here strongly support targeting HTT1a in the design of therapeutic approaches to lower huntingtin.

## Supplementary Material

awag040_Supplementary_Data

## Data Availability

RNA sequencing data have been deposited in GEO with the accession number GSE310158. Nanopore data have been deposited in GEO with the accession number GSE307808. The authors confirm that all other data supporting the findings in this study are available within the article and its Supplementary material. Raw data will be shared by the corresponding author upon request.
