# Supplementary material for: The HTT1a protein initiates HTT aggregation in a knock-in mouse model of Huntington’s disease
Source: Brain. 2026 Feb 2;149(9):3095–109. doi: 10.1093/brain/awag040 (PMC13548859; doi:10.1093/brain/awag040)
Supplement: Supplementary file 1 [file awag040_supplementary_data.pdf]

SUPPLEMENTARY MATERIAL

The HTT1a protein initiates HTT aggregation in a knock-in mouse model of Huntington's disease

Aikaterini Smaragdi Papadopoulou[1], Christian Landles[1†], Edward J. Smith[1†], Marie K. Bondulich[1], Annett Boeddrich[2], Maria Canibano-Pico[1], Emily C. E. Danby[1], Franziska Hoschek[3], Arzo Iqbal[1], Samuel T. Jones[1], Nancy Neuendorf[2], Iulia M. Nita[1], Georgina F. Osborne[1], Jemima Phillips[1], Maximilian Wagner[3], Erich E. Wanker[2], Jonathan R. Greene[4], Andreas Neueder[3,5], Gillian P. Bates[1]

[†]These authors contributed equally to this work.

[1]Huntington's Disease Centre and Department of Neurodegenerative Disease, Queen Square Institute of Neurology, UCL; London WC1N 3BG, UK.
[2]Neuroproteomics, Max Delbrueck Center for Molecular Medicine, 13125 Berlin, Germany.
[3]Department of Neurology, University Hospital Ulm, 89081 Ulm, Germany.
[4]Rancho BioSciences; San Diego, California 92127, USA.
[5]University Medical Center Hamburg-Eppendorf, Center for Molecular Neurobiology, 20251 Hamburg, Germany.

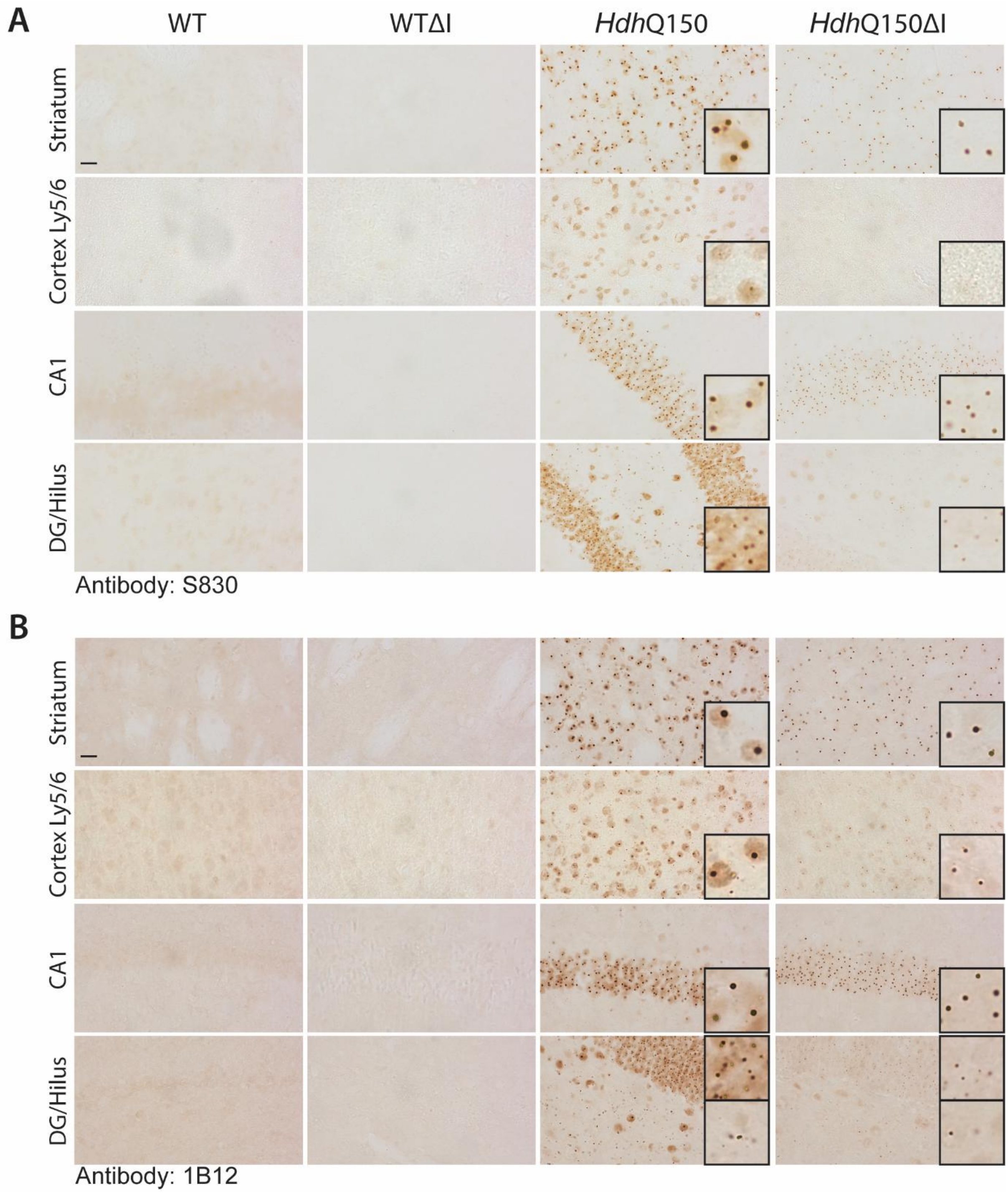

Supplementary Figure 1. The S830 and 1B12 antibodies do not detect an aggregate signal in either WT or WTΔI sections. Immunohistochemistry with the (**A**) S830 and (**B**) 1B12 antibodies to brain sections from WT, WTΔI, *Hdh*Q150 and *Hdh*Q150ΔI mice at 17 months of age. Scale bar = 20 μm, zoomed images are 20 μm^2. DG = dentate gyrus, Ly = layers, WT = wild type.

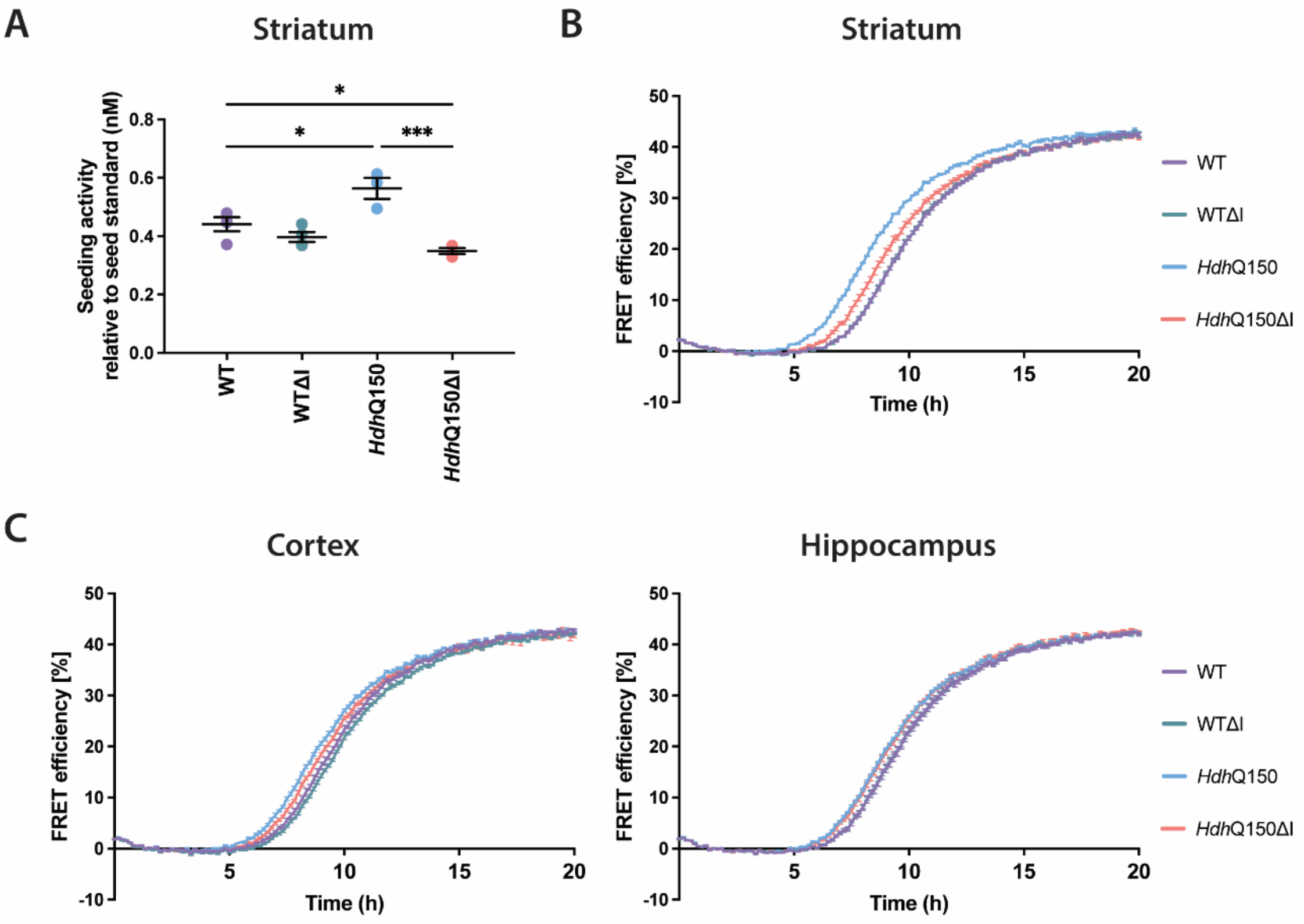

Supplementary Figure 2. HTT aggregate seeding activity is present in *Hdh*Q150 striatum at 6 months of age. (**A**) Quantification of mutant HTT seeding activity in the striatum of WT, WTΔI, *Hdh*Q150 and *Hdh*Q150ΔI mice at 6 months of age. Only the *Hdh*Q150 striatum gave a signal above background; *n* = 3-4 / genotype. (**B**) Effect of striatal homogenates from the WT, WTΔI, *Hdh*Q150 and *Hdh*Q150ΔI mice at 6 months of age on the Ex1Q48-CyPet and -YPet (1:1 mixture, 1.2 nM) co-aggregation; *n* = 4 / genotype. (**C**) Effect of cortical and hippocampal homogenates from the WT, WTΔI, *Hdh*Q150 and *Hdh*Q150ΔI mice at 6 months of age on the Ex1Q48-CyPet and -YPet (1:1 mixture, 1.2 nM) co-aggregation; *n* = 4 / genotype. Statistical analysis was one-way ANOVA with Tukey's post hoc correction. Error bars: mean ± SEM. **P* ≤ 0.05, ****P* ≤ 0.001. h = hours, WT = wild type.

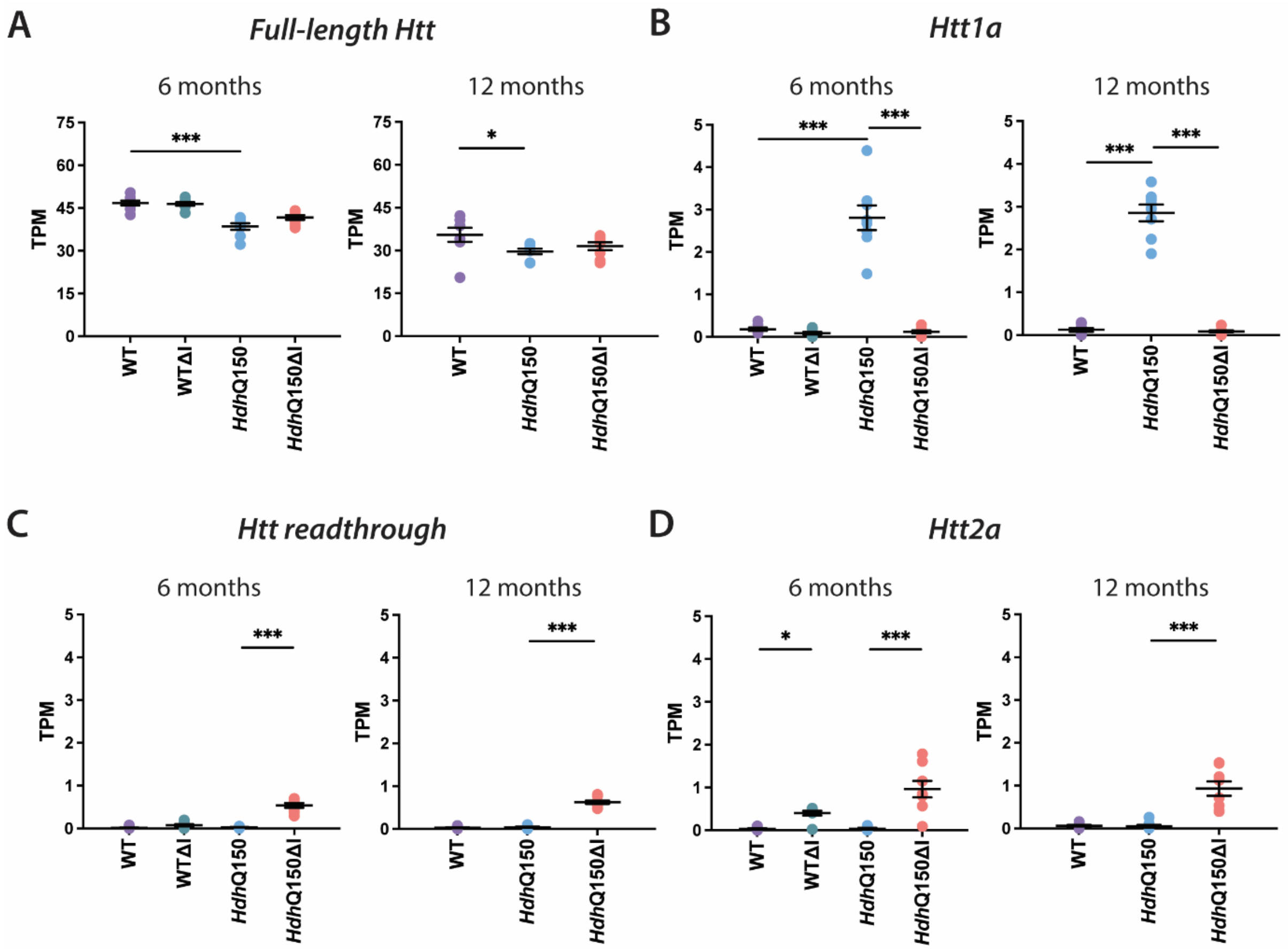

Supplementary Figure 3. Hippocampal huntingtin transcripts generated from the genetically modified loci. (**A-D**) Quantification of (**A**) the full length *Htt* transcript (**B**) the *Htt1a* transcript (**C**) the *Htt readthrough* transcript and (**D**) the *Htt2a* transcript in WT, WTΔI, *Hdh*Q150 and *Hdh*Q150ΔI hippocampus at 6 months of age and WT, *Hdh*Q150 and *Hdh*Q150ΔI hippocampus at 12 months. $n = 8$ / genotype, equal sexes. Statistical analysis was by DESeq2 with Benjamini-Hochberg correction. Error bars: mean ± SEM. $^{*}P \leq 0.05$, $^{***}P \leq 0.001$. TPM (transcripts per million) were computed from RNA-seq data at the indicated ages. WT = wild type.

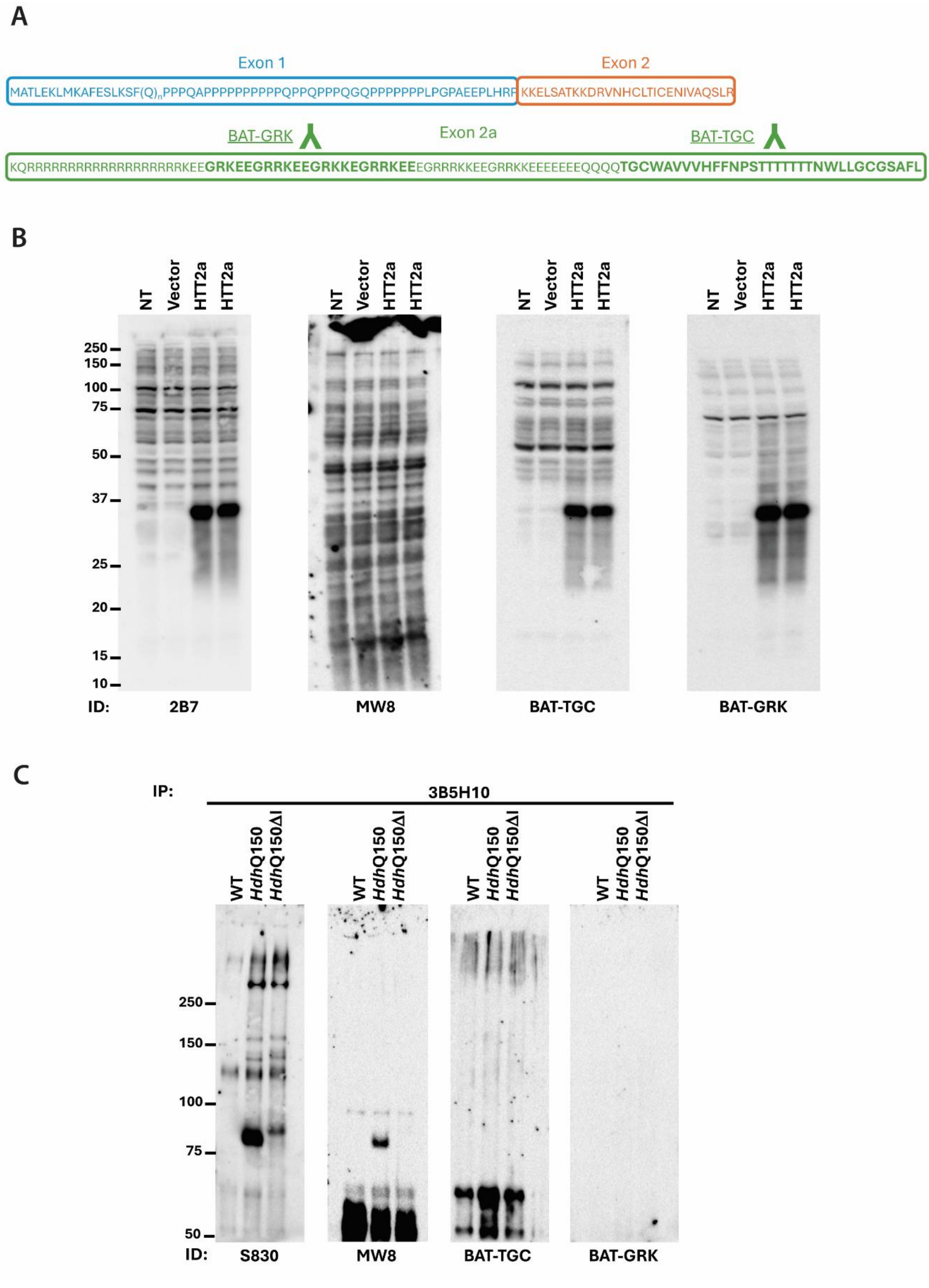

Supplementary Figure 4. The HTT2a protein could not be detected in the brains of *Hdh*Q150ΔI mice. (**A**) The predicted sequence of the HTT2a protein and position of the sequences used to raise the BAT-GRK and BAT-TGC antibodies. (**B**) Western blot of lysates from COS-1 cells transfected with a *Htt2a* construct fractionated on a 12% SDS-PAGE gel. The HTT2a protein is detected by the 2B7 (amino acids 7-12), BAT-GRK and BAT-TGC

antibodies, but not by the HTT1a specific antibody, MW8. (**C**) Mutant HTT proteins immunoprecipitated with the 3B5H10 antibody from brain hemispheres of WT, *Hdh*Q150 and *Hdh*Q150ΔI mice at 2 months of age and fractionated on a 10% SDS-PAGE gel. The western blot was immunoprobed with S830, MW8, BAT-GRK and BAT-TGC. S830 detects full-length HTT, its proteolytic fragments and HTT1a and MW8 detects HTT1a. The BAT-GRK and BAT-TGC antibodies do not detect any bands specific to the *Hdh*Q150ΔI brains that could correspond to HTT2a. NT = non-transfected, transfection reagents only, vector = empty vector. Size markers are in kDa.

**Founder**	**Sex**	**Deletion**	**Genomic coordinates**	**Selected**
**#113**	F	19998 bp	722-20719	
**#123**	M	19998 bp	722-20719	x
**#130**	F	19997 bp	723-20719	
**#142**	F	19997 bp + 10 bp insertion	723-20719	
**#146**	M	20002 bp	721-20722	x
**#220**	F	20010 bp	713-20722	
**#242**	M	20003 bp + 4 bp insertion aact	721-20723	

Supplementary Table 1. Summary of the nature of the intron 1 deletion on the WT allele from the first genetic modification experiment. Colonies were established from lines #123 and #146 and line #123 was used as the WTΔI line.

Name	Immunogen	Epitope	Species	Source
**2B7**	Human HTT peptide: aa 1-17[1]	LMKAFE*	Mouse monoclonal	CHDI Foundation
**MW1**	GST-DRPLA (19Q)[2]	PolyQ	Mouse monoclonal	CHDI Foundation
**3B5H10**	GST-HTT N171 (66Q)[3]	PolyQ	Mouse monoclonal	Sigma-Aldrich P1874
**S830**	GST-human exon 1 HTT (53Q)[4]	N/A	Sheep polyclonal	In house
**CHDI-90001414**	Mouse proline-rich regions of HTT1a	Mouse polyP region	Rabbit polyclonal	CHDI Foundation
**MW8**	Human exon1 HTT (67Q)[2]	C-terminus of HTT1a[5]	Mouse monoclonal	CHDI Foundation
**CHDI-90000148**		C-terminus of HTT1a	Rabbit polyclonal	CHDI Foundation
**P90 clone: 1B12**	AEEPLHRP-OH	C-terminus of HTT1a	Rabbit monoclonal	CHDI Foundation
**AB2644**	Human exon 2 HTT	N/A	Rabbit polyclonal	This publication
**MAB5490**	Human HTT: aa 115-129[6]	Within: QSVRNSPEFQKLLGI (mouse L)	Mouse monoclonal	Sigma-Aldrich, MAB5490
**MAB2166**	HTT fusion protein: aa 181-810[7]	Within aa 443-457 GKVLLGEEEALEDDS [8]	Mouse monoclonal	Sigma-Aldrich, MAB2166
**D7F7**	HTT peptide	Within: aa 1214-1223* QSDTSGPVTT (mouse A)	Rabbit monoclonal	Cell Signaling Technology #5656
**BAT-GRK**	GRKEEGRRKEEGRKKEGRRKEE	N/A	Rabbit polyclonal	This publication
**BAT-TGC**	TGCWAVVVHFFNPSTTTTTTTNWLLGCGSAFL	N/A	Rabbit polyclonal	This publication

*Information provided by the CHDI Foundation

Supplementary Table 2. Summary and source of the antibodies used for western blotting, immunohistochemistry and the HTRF assays. DRPLA = dentatorubral-pallidoluysian atrophy, polyQ = polyglutamine, polyP = polyproline.

Antibody Pairing	HTT isoform	Donor (ng / well)	Acceptor (ng / well)	Lysate Concentration
**D7F7-Tb:MAB5490-d2**	Full-length HTT	1 ng	40 ng	5%
**MAB5490-Tb: MW1-d2**	Mutant HTT	1 ng	40 ng	5%
**2B7-Tb: MW8-d2**	HTT1a	1 ng	40 ng	10%
**MW8-Tb: CHDI-1414-d2**	HTT aggregation	1 ng	40 ng	10% Crude Lysate

Supplementary Table 3. Summary of the antibody concentrations and lysate dilutions used for the HTRF assays.

Ensembl	Gene	Mean	AbsLog2FC	Log2FC	Stat	P value	AdjP	Log2FCSE
ENSMUSG00000068876	*Cgn*	102.17	0.56	0.56	5.15	2.58E-07	3.17E-03	0.11
ENSMUSG00000062078	*Qk*	7475.94	0.18	-0.18	-5.23	1.65E-07	3.17E-03	0.03
ENSMUSG00000042133	*Ppig*	2315.00	0.19	-0.19	-5.07	3.98E-07	3.27E-03	0.04
ENSMUSG00000020570	*Sypl*	1102.43	0.23	-0.23	-4.88	1.04E-06	6.40E-03	0.05
ENSMUSG00000024411	*Aqp4*	1931.38	0.27	-0.27	-4.77	1.81E-06	8.88E-03	0.06
ENSMUSG00000031578	*Mak16*	555.82	0.22	-0.22	-4.62	3.89E-06	1.59E-02	0.05
ENSMUSG00000019966	*Kitl*	859.38	0.29	-0.29	-4.45	8.45E-06	2.45E-02	0.06
ENSMUSG00000068798	*Rap1a*	1058.84	0.22	-0.22	-4.44	8.95E-06	2.45E-02	0.05
ENSMUSG00000093989	*Rnasek*	1746.65	0.12	0.12	4.45	8.53E-06	2.45E-02	0.03
ENSMUSG00000022558	*Mroh1*	1144.99	0.21	0.21	4.34	1.42E-05	3.49E-02	0.05
ENSMUSG00000063954	*H2ac19*	58.86	7.20	7.20	4.30	1.72E-05	3.52E-02	1.68
ENSMUSG00000022899	*Slc15a2*	410.13	0.35	-0.35	-4.31	1.60E-05	3.52E-02	0.08
ENSMUSG00000028100	*Nudt17*	38.60	0.70	0.70	4.24	2.21E-05	3.87E-02	0.17
ENSMUSG00000024974	*Smc3*	1604.73	0.14	-0.14	-4.25	2.18E-05	3.87E-02	0.03

Supplementary Table 4. List of genes that are differentially expressed between WTΔI and WT striata at 6 months of age. Differential expression between the WTΔI and WT striatal RNA-seq data sets from 6-month-old mice at a significance threshold of an adjusted $P < 0.05$ after multiple-test correction, and fold changes of at least 20% in either direction. AbsLog2FC = absolute $\log^2$ fold change, Log2FC = directional $\log^2$ fold change; a negative value indicates decreased expression in WTΔI mice, Stat = Wald statistic, AdjP = false discovery rate adjusted P value, Log2FCSE = standard error of the $\log^2$ fold change.

HD Signature	HD Signature	Reversed	% Reversed	Full Reversal	Partial Reversal	Super Reversal	Exacer-bation	% Exacer-bation
***Hdh*Q150ΔI – 6 m Striatum**	1254	314	25	86	228	0	6	0
***Hdh*Q150ΔI – 6 m Hippocampus**	61	18	30	5	13	0	0	0
***Hdh*Q150ΔI – 12 m Striatum**	2821	1141	40	461	663	17	14	0
***Hdh*Q150ΔI – 12 m Hippocampus**	633	331	52	123	207	1	0	0

Supplementary Table 5. Change in the numbers of dysregulated transcripts in the *Hdh*Q150ΔI striatum as compared to *Hdh*Q150. M = months.

References

1. Weiss A, Abramowski D, Bibel M, Bodner R, Chopra V, DiFiglia M, Fox J, Kegel K, Klein C, Grueninger S, Hersch S, Housman D, Regulier E, Rosas HD, Stefani M, Zeitlin S, Bilbe G, Paganetti P. Single-step detection of mutant huntingtin in animal and human tissues: A bioassay for Huntington's disease. *Anal Biochem*. Dec 1 2009;395(1):8-15. doi:S0003-2697(09)00541-7
2. Ko J, Ou S, Patterson PH. New anti-huntingtin monoclonal antibodies: implications for huntingtin conformation and its binding proteins. *Brain Res Bull*. Oct-Nov 1 2001;56(3-4):319-329.
3. Peters-Libeu C, Newhouse Y, Krishnan P, Cheung K, Brooks E, Weisgraber K, Finkbeiner S. Crystallization and diffraction properties of the Fab fragment of 3B5H10, an antibody specific for disease-causing polyglutamine stretches. *Acta Crystallogr Sect F Struct Biol Cryst Commun*. Dec 1 2005;61(Pt 12):1065-1068. doi:S1744309105036547
4. Sathasivam K, Woodman B, Mahal A, Bertaux F, Wanker EE, Shima DT, Bates GP. Centrosome disorganization in fibroblast cultures derived from R6/2 Huntington's disease (HD) transgenic mice and HD patients. *Hum Mol Genet*. 2001;10(21):2425-2435.
5. Landles C, Sathasivam K, Weiss A, Woodman B, Moffitt H, Finkbeiner S, Sun B, Gafni J, Ellerby LM, Trottier Y, Richards WG, Osmand A, Paganetti P, Bates GP. Proteolysis of mutant huntingtin produces an exon 1 fragment that accumulates as an aggregated protein in neuronal nuclei in Huntington disease. *J Biol Chem*. Mar 19 2010;285(12):8808-8823. doi:M109.075028
6. Lunkes A, Lindenberg KS, Ben-Haiem L, Weber C, Devys D, Landwehrmeyer GB, Mandel JL, Trottier Y. Proteases acting on mutant huntingtin generate cleaved products that differentially build up cytoplasmic and nuclear inclusions. *Mol Cell*. Aug 2002;10(2):259-269.
7. Trottier Y, Devys D, Imbert G, Saudou F, An I, Lutz Y, Weber C, Agid Y, Hirsch EC, Mandel JL. Cellular localization of the Huntington's disease protein and discrimination of the normal and mutated form. *Nat Genet*. 1995;10(1):104-110.
8. Cong SY, Pepers BA, Roos RA, Van Ommen GJ, Dorsman JC. Epitope mapping of monoclonal antibody 4C8 recognizing the protein huntingtin. *Hybridoma (Larchmt)*. Oct 2005;24(5):231-5. doi:10.1089/hyb.2005.24.231
